# Development of an interdisciplinary pre-matriculation program designed to promote medical students’ self efficacy

**DOI:** 10.1080/10872981.2017.1272835

**Published:** 2017-01-09

**Authors:** Anna Wirta Kosobuski, Abigail Whitney, Andrew Skildum, Amy Prunuske

**Affiliations:** ^a^ Department of Biomedical Sciences, University of Minnesota Medical School, Duluth Campus, Duluth, MN, USA; ^b^ University of Minnesota, Duluth, MN, USA

**Keywords:** Under-represented students, microbiology, rural, Native American, pre-matriculation

## Abstract

**Background and objectives:** A four-week interdisciplinary pre-matriculation program for Native American and rural medical students was created and its impact on students’ transition to medical school was assessed. The program extends the goals of many pre-matriculation programs by aiming to increase not only students’ understanding of basic science knowledge, but also to build student self-efficacy through practice with medical school curricular elements while developing their academic support networks.

**Design:** A mixed method evaluation was used to determine whether the goals of the program were achieved (n = 22). Student knowledge gains and retention of the microbiology content were assessed using a microbiology concept inventory. Students participated in focus groups to identify the benefits of participating in the program as well as the key components of the program that benefitted the students.

**Results:** Program participants showed retention of microbiology content and increased confidence about the overall medical school experience after participating in the summer program.

**Conclusions:** By nurturing self-efficacy, participation in a pre-matriculation program supported medical students from Native American and rural backgrounds during their transition to medical school.

**Abbreviations:** CAIMH: Center of American Indian and Minority Health; MCAT: Medical College Admission Test; PBL: Problem based learning; UM MSD: University of Minnesota Medical School Duluth

## Introduction

There has been an increased popularity of pre-matriculation courses in the nation’s medical schools as a means to reduce student academic hardship and attrition [1]. These programs vary significantly in length, activities, and content. Some programs expose students to a particular subject area thereby decompressing the first year of school whereas others center on development of skills such as patient communication or promoting connections to rural communities [2]. Though well regarded anecdotally, the direct benefits have not been rigorously analyzed.

At the University of Minnesota Medical School Duluth campus (UM MSD) we chose to design a program beginning with the view that we would extend beyond goals of many programs by placing greater emphasis on student self-efficacy as a means to promote student success throughout their medical education. The UM MSD mission is to educate students who will practice in Minnesota and Native American communities. Both rural and Native American communities are vastly underserved [3,4]. UM MSD recognizes these needs and is known for its adherence to its social mission.

Students from under-represented minority (American Indian/Alaska Native, African American, Hispanic or Latino) and rural backgrounds have an increased likelihood of serving communities like their own [5–8]. Thus, it is incumbent on medical schools to address the academic needs of these students early on; failure to do so becomes increasingly problematic over time and can result in needlessly delaying graduation and even failure to graduate [9]. The human and economic costs are considerable and are reflected in lost time, social and personal stress and considerable financial burden for both the individual and the institution. While UM MSD has an excellent record of student retention, including that of Native American and disadvantaged rural students, a central goal of our program is to improve self-efficacy by fostering student confidence and a belief that they have the capabilities to be successful in medical school [10]. The development of self-efficacy is associated with completion of mastery experiences and leads to resilience in the face of failure, development of deep learning approaches, and maintenance of emotional well-being [11]. Such self-assuredness aids in reducing remediation and its many associated costs.

Rural and Native American students share a number of obstacles that can hinder their access to and persistence in medical education. Though they most often come from families and communities that support and encourage their pursuit of advanced education [12,13], both of these student populations also experience significant life challenges including lack of requisite academic preparation and guidance, lower socioeconomic status, fewer role models, and fewer social supports [14–16]. Native American communities may further harbor a distrust of academic institutions cultivated by a history of removal of young children to federal boarding schools and unethical past and present research practices [17,18]. In the college environment, Native American students may further experience loss of cultural connectedness, distancing from family, bias, stereotyping, and isolation [12,19–21]. For students facing this complex myriad of barriers, the realization of a medical education can seem implausible.

Medical schools may choose to employ holistic review of their applicants in order to fulfill medical school missions and better ensure diversity in our physician workforce. As such, admission committees ‘might consider a wide range of factors, including race, gender, socioeconomic status, educational background, languages spoken, and geographic origin’ [22]. Rural and Native American students may be considered in light of any number of these areas. Taking the example of socioeconomic status, Grbic, Jones, and Chase [23] found that parental education and occupation are reliable indicators. The rural populous has lower academic achievement, works in occupation classifications that require less formal education, and earn a lower income [24]. Native Americans, along with having the highest poverty rate of all race and ethnic groups [25], share the nation’s highest unemployment rate with African Americans [26], and have the lowest educational attainment rate of any race or ethnic group [27].

UM MSD’s Pre-Matriculation Program was created based on the curricular and social needs of our students. The curriculum design emulates UM MSD’s pedagogic approaches and reflects the feedback of faculty and former students regarding areas they would most appreciate given their medical education perspectives and experiences. By doing so, we were assured that the program components attended to the students’ scholastic and social needs that are most critical to successful academic progression. We set out to promote student success by immersing them in a rigorous four-week holistic medical school experience that includes coursework, weekly exams, and problem-based learning as well as enrichment activities including laboratory sessions, journal club, and grand rounds to aid in developing critical thinking, public speaking, and student confidence. We further employed intervention strategies to aid in skill development, self-confidence, and familiarization with medical school resources and processes critical for successful advancement toward graduation. Importantly, our decision to include small cohorts of students allows them to forge supportive personal and cultural support networks.

We have evaluated our program over the course of three years using a mixed method approach to look at the following questions: (1) What knowledge and skills do students gain through participation in the program? (2) What components of the program were critical to achieving these competencies?

## Methods

### Institutional context

The University of Minnesota Medical School Duluth campus (UM MSD) is a regional campus with a social mission to train physicians who will serve rural Minnesota and Native American communities. UM MSD is recognized for our 40-year history of successfully fulfilling our mission. Our school is ranked fifth in rural medicine, eleventh in family medicine, and ninth in primary care [28]; we have also graduated the second largest number of Native American physicians [29]. These outcomes are attributable to the school’s mission. Our compact building and small class size of 60 students help facilitate peer and student/faculty relationships and ready access to resources. Students complete their first two years of basic science education in Duluth, then transfer to the Twin Cities to complete their third and fourth years of clinical training. The school is home to the Center of American Indian and Minority Health (CAIMH) which has played an integral role in Native American medical student recruitment and retention. CAIMH efforts include a health professions pipeline for K-16 Native American students and provides personal and cultural support for medical students. Noteworthy is that approximately 16% of the UM MSD faculty are Native American (vs. 0.13% nationally) [30]; one of the faculty is also an Associate Dean for Curriculum and Medical Education. Moreover, a number of the school’s entire faculty have health disparity focused research interests.

### Pre-matriculation program overview

The UM MSD Pre-Matriculation Program has been offered to students in its current form since 2013. A limited number of admitted students are invited to participate in the program. The project was funded by a Department of Health and Human Services Health Resources and Services Administration (HRSA) grant and the criteria for participation are in accordance with those set forth by the agency (http://bhpr.hrsa.gov/grants/diversity/hcop.htmlwere). Supplemental funds have been provided by the UM MSD Dean’s Office to support the inclusion of students who were deemed academically at-risk (science GPA below 3.6 and/or MCAT below 27), but do not meet the HRSA’s educationally disadvantaged definition. Though the HRSA funding ended after the 2015 program the school saw its value; the 2016 program was funded in part by CAIMH and in part by the UM MSD Dean’s Office, with plans to continue future years. For the first two years, participants included only incoming students; in 2015, we expanded to include students who were repeating first year courses. The program occurs on the UM MSD campus; students receive a living allowance and funds to help cover travel and housing.

### Pre-matriculation program curriculum

**Figure 1.**
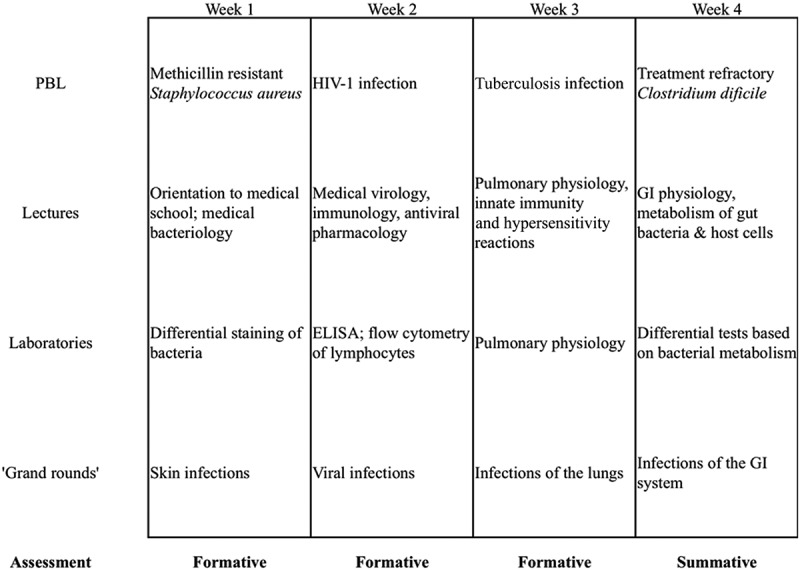
Curricular elements of a four-week retention program for incoming medical students. Curricula was designed to combine active learning and more standard elements, and included weekly problem based learning (PBL) cases, didactic lectures, laboratories, and student presentations of a clinical infectious disease case (‘Grand rounds’), (ELISA, enzyme linked immunosorbant assay; GI, gastrointestinal; HIV-1, human immunodeficiency virus −1).

The UM MSD Pre-Matriculation Program consists of a four-week course in the summer immediately prior to the beginning of the fall semester. The course is divided into weekly blocks (Figure 1), each centered around a problem based learning (PBL) case that focuses on an infectious disease and integrates many other medical disciplines. For each weekly block, students participate in PBL for five hours. Eight to ten hours per week are devoted to didactic lecture with topics that include physiology, biochemistry, microbiology, pharmacology, immunology, and clinical practice. Four hours per week students conduct laboratories that focus on clinical science applications of basic science principles. Each week students also research an assigned infectious disease case and give fifteen minute oral presentations to their peers and course faculty in a ‘Grand Rounds’ session.

Each week one hour is allocated for formative assessment consisting of multiple choice questions written by course faculty and administered through the same computerized systems used by all students throughout the academic year curriculum. After the assessments, a review ensures student comprehension of subjects they scored poorly in and a chance to discuss test taking strategies. At the conclusion of the four-week course, a comprehensive final examination is administered, and each student’s assessment performance is reviewed one-on-one with course faculty.

Importance is placed on establishing strong social and professional networks throughout the program, and there are frequent lunches with faculty and local clinicians. We have further implemented a formal peer mentorship component in which two second-year students go through the curriculum with program participants and help them to develop appropriate study techniques. The course concludes with a picnic meal prepared by course faculty.

### Human subjects review

This study was approved as exempt research by the University of Minnesota IRB (#1306S36782) and included 22 UM MSD retention program participants between 2013–2015. Participants had the choice to decline participation in the end of program survey and focus group. All data were de-identified during analysis.

### Concept inventory

On the first day of the UM MSD Pre-Matriculation Program, participants took the multiple-choice Host Pathogen interaction concept inventory test [31]. A week and a half after the end of the program during medical school orientation, all of the incoming medical students completed the same concept inventory test. The test was administered online using the medical school testing software. Basic descriptive statistics and graphs were compiled in Excel. A paired t-test of the concept inventory was completed using JMP to compare individual changes and an unpaired t-test was completed for group differences (http://www.jmp.com/en_us/software.html).

### Focus group

The qualitative data for this study were derived from focus groups conducted after the participants had completed their first semester of medical school. All consented to participation and were given the option not to respond to certain questions or to withdraw from the group at any time. The focus group script is in the appendix; there were slight revisions to the script each project year to reflect program modifications that were made based on student feedback. The interviews were conducted in groups of 2–6 participants at the UM MSD by student project assistants who were not involved in the delivery of the Pre-matriculation program curriculum. Students were asked specifically about their impressions of curriculum delivered online and whether an online course could have been as effective as a program delivered in person. Sessions were digitally-recorded to ensure accuracy; recordings were transcribed verbatim, and student identity was removed.

Rigor in the thematic analysis was ensured through a deliberate process. Using a constant comparative method of analysis [32] Researcher 1, Researcher 2, and Researcher 3 separately read each of the de-identified transcripts to gain an overall sense of each session and participant discussion; they later convened to discuss their impressions. Researchers 1 and 2 separately hand-coded the transcripts to identify general themes using both hand-written notes and tracking comments on Microsoft Word documents. Researchers 1 and 2 then reconvened to discuss, resolve any differences in coding, clarify concepts, and refine until consensus was reached. Researcher 1 re-examined transcripts line-by-line to ensure that codes accurately reflected the intent of the participants’ responses. The process was deemed complete as Researchers 1 and 2 again met, discussed and agreed on the accuracy of the analysis. Excerpts presented in the Results section are identified by a participant number and year of program participation (e.g. P1, 2014); when there were two separate focus groups, a group number is also included (e.g., P1, Grp 2, 2015). The 2013 transcripts did not include unique participant identifiers, only a generic identifier for all group participants (GP, 2013) and limits the ability to discern whether responses were on the part of a single articulate participant or across several individuals in the group.

## Results

### Pre-matriculation program development

UM MSD recognizes its institutional responsibility to support and retain rural and Native American medical students during their basic science years with the acknowledgment this demands institutional investment and faculty buy-in. We maintain high expectations for all students, reflect on hidden curriculum, and work to recognize and address underlying societal factors contributing to students’ preparation for medical school.

Acknowledging playing fields are not level, we identify and invite a subset of academically at-risk students to participate in a pre-matriculation program prior to entrance to medical school. The most recent cohorts are those we included in this study and were comprised of six students in 2013; three in 2014; and 13 (including two students who were retaking first year courses) in 2015. The participants as compared to the overall class are more likely to be under-represented minorities, older, women, and from out-of-state (Table 1).

**Table 1.** Demographics of program participants.

	Pre-matriculation participants (N=22)		Entire matriculating Class (N=180)	
Under-represented minority	72.7%		11.1%	
Disadvantaged SES mtatus (2014 and 2015 only)	43.8%		33.3%	
Rural hometown (<20 000)	59.1%		82.8%	
Minnesota resident	45.5%		91.1%	
Average age at matriculation	26		23	
Women	63.6%		49.4%	
		Range		Range
Average MCAT	26.04	24–32	28.84	23–36
MCAT BS	9.09	7–11	10.08	7–14
MCAT PS	6.41		9.43	5–14
MCAT V	6.14		9.33	5–13
BCPM	3.27	1.93–3.98	3.61	1.93–4.00

Between 2005–2009, the school was home to another pre-matriculation program which focused on delivering histology and pathology content prior to entry. After a move toward a problem and system based curriculum, we redesigned the pre-matriculation program to our current interdisciplinary, pre-matriculation program based on input of former students and faculty regarding student academic needs. We first developed a survey and administered it to current medical students to identify areas of need, and a team of four faculty with expertise in training preclinical medical students developed the new curriculum (Figure 1). These faculty were on nine-month appointments and did receive salary to cover summer months, however, this meant that they were not able to dedicate that time to lab research that is a school expectation.

An infectious disease theme was selected because we found that 50% of our students had not taken a microbiology course and 75% had not had not taken an immunology course. We further integrated several other disciplines including biochemistry, physiology, and pharmacology. The curricular elements allowed for exposure similar to the first year curriculum such as faculty lectures and a weekly problem-based learning case. The small group size permitted supplemental microbiology labs and student case presentations. Lectures were delivered by a core group of faculty whose academic year teaching load included portions of the introductory first-year basic science course. We strategically invited faculty experts to lead blocks of instruction to further envelope the students in the medical school environment. Weekly formative assessments and one final exam furnished students with feedback on their attainment of the course learning objectives, practice with the multiple-choice question format and computerized testing system. On the end of course survey, students rated each of the program components highly. Student feedback each year has helped us continuously improve the program by taking steps such as improving peer mentor study sessions and implementing a journal club session.

### Concept inventory

**Figure 2.**
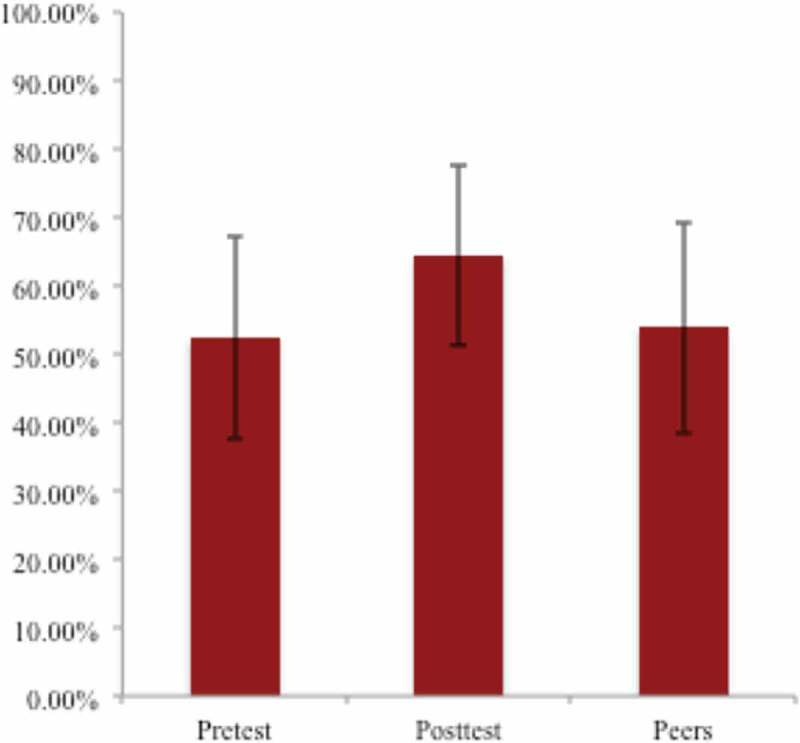
Microbiology concept inventory. Students in the pre-matriculation program were administered the Host Pathogen Interactions concept inventory at the beginning of the program (Pretest). All medical students took the exam during orientation week, which was a week and half after the end of the program. The average scores and standard deviation were calculated for participants in the program (Posttest) and nonparticipants (Peers). The difference between the pretest and posttest was significantly different by a paired t-test (p = 0.0002, n = 22).

We administered the Host Pathogen Interaction concept inventory before the course and two weeks after the end of the course to assess student retention of the program’s microbiology objectives. Concept inventories are validated tests and can be useful tools to ascertain whether a learner is grasping big picture concepts [33]. After comparing results of each individual’s pre-test and post-test, participants did in fact make gains in key microbiology concepts, scoring 52.4% on the pre-test and 64.4% on the post-test (Figure 2, p = 0.0002). The gains between years was not significantly different. These results suggest that the four-week program indeed increased students’ comprehension and retention of microbiology content essential to adequate performance in medical school coursework.

### Focus group

We chose to include a qualitative component to the study to complement our quantitative findings to provide the benefit of added richness and depth to our description of the program participants’ experiences. We conducted semi-structured focus groups following the first semester of medical school, all of the pre-matriculation program participants chose to take part with the exception of two students in the 2015 group. These groups gave each student an opportunity to reflect on whether the program adequately prepared them for the challenges presented by their coursework. From these groups there were three major themes that emerged: (1) Anxiety about transition to medical school; (2) Study skills, time management, and faculty access; (3) Development of peer relationships.

We found that becoming familiar with the school and curriculum, and being non-traditional students were primary motivators for participation in the pre-matriculation program. Participants commonly cited anxiety about transitioning to medical school (as related to academics, self-confidence, managing home life). A 2013 participant noted, ‘I was very apprehensive about not being able to cut it [in medical school]. So when they asked if I wanted to do this program it was just a no brainer.’ Immersion in the medical school experience allowed students to smoothly transition to and increase comfort and confidence in the new setting: it helped ‘me gain my bearings at the school’ (P2, 2014). The program provided the opportunity to sample course content, teaching formats, assessments using the same testing software, and become familiar with resources and supports available in their upcoming first and second year, ‘I liked the resources they kind of walked us through’ (GP, 2013). Participants were also able to resolve uncertainties about how to effectively strike a school and life balance, ‘I could see the level of effort that I needed to put into it while still being able to meet my other responsibilities’ (P1, 2014). The participants each year agreed that the program alleviated anxiety and increased confidence in the medical school environment; in the words of one participant, ‘oh my gosh, I can actually do this’ (P1, Grp 2, 2015).

Developing effective study and time management skills prior to the onset of the demanding medical school coursework was identified as an unqualified program plus: ‘it gave me… an opportunity to try a different study method. I completely changed my system from what I did in undergrad’ (GP, 2013). As another put it,

I realized that in order for me to learn, I have to figure out how to draw something, or how to interact with the material in a way that would make it stick the first or the second time I look at it. (P2, 2014)

A common retention barrier is ensuring that students access help in their courses before soluble issues swell to academic hardship or failure. Interaction with the program faculty and learning to use them as a resource was viewed as particularly beneficial in this regard and carried over into the academic year. For one student, ‘when I struggled, it made me feel like there was already someone on my team ’ (P3, Grp 2, 2015). Upon reflection, participants recognized that the competencies they developed during the program were indeed critical skillsets when they tackled the academic year curriculum: ‘I wasn’t as nervous as a lot of other people who didn’t have this for our first exam’ (P4, Grp2, 2015).

Likewise, the participants found the occasion to build supportive peer relationships rewarding in assorted ways. Most apparent were the social bonds found in the formation of peer relationships, ‘some of my closest friend would be ones I met [in the program]’ (P4, Grp 1, 2015). An outgrowth of these connections was the participants found another outlet for asking for help when needed, ‘it made working with other students a lot easier for me. I’m not afraid to go ask someone’ (P1, Grp 1, 2015).

An area we have struggled to balance is how much content to include in the program curriculum. In general, we have structured the program so participants can also get used to the demands associated with the medical curriculum. However, the first course taken is very lecture heavy and the summer program incorporates more active learning elements along with lecture. Consequently, some students felt their summer experience was not reflective of the intensity of expectations required during the semester. Nevertheless, when asked if they would like increased rigor and lecture content, the response was mixed. Some liked the idea of closely mimicking the school year to get a precise grip on what was to come. Others, especially those relocated from other regions or who had families, placed greater importance on having the option of getting settled in. One expressed ‘you’re already moved in and unpacked and you know your way around town a little bit, and that’s just one less thing to be stressed about’ (P2, Gr 1, 2015).

In the end, the students were uniformly positive about and grateful for their experience with the program. They further unanimously agreed that the program has been an essential element to their successful first year of medical school and would highly recommend it to others.

## Discussion

Pre-matriculation courses are a viable strategy to support the training of a diverse set of physicians who may come from educationally disadvantaged backgrounds. Thus, it is vital that existing programs identify the effective strategies and local factors that facilitate such desired outcomes as retaining admitted students. The UM MSD has an accomplished history of cultivating an environment where students from rural and Native American backgrounds may thrive. Our pre-matriculation program mirrors our school’s preventive medicine focus by promoting the confidence, skills, and connections students need upfront as a means of minimizing hardship during their medical training.

Incorporating online elements into pre-matriculation programs is becoming more common. Admittedly, this can be a cost-effective way to reach larger numbers of students and disseminate foundational content [34,35]. However, we found that this strategy would undermine essential components of our program such as development of a rounded skillset, building necessary social connections and familiarity with place. The question of the efficacy of online programming for our purposes was met by an unequivocal ‘No’ from our focus group participants.

Some programs choose a design that consists of a reduced length and a format generally comprised of a basic overview of resources and very little (or no) time for students to become acquainted with, develop, practice, and ultimately adopt the skills and behaviors essential to their medical education. Conversely, our approach of immersion in the basic science curriculum and weekly exams with the expectation of a passing score encouraged students to experiment with study strategies and explore the sometimes tricky balance of time management to discover what was effective and best suited their individual needs. An abbreviated program structure would also preclude interpersonal interaction with and role modeling by school faculty and administration. Faculty and student relationships were further nurtured as the small group size allowed for elevated interaction during didactic lectures than is possible with the entire student body. These relationships fostered comfort in the confidence to ask for help or clarification, an obstacle that for some students can wholly decide passing or failing a course. Importantly, because the homes of our students were geographically diverse, our extended program allowed them to establish themselves (and in some cases, their families) and become accustomed to the culture of the region and school through a non-threatening process. The transition to a new school can be stressful and having the benefit of time prior to the school year alleviated stress, thus allowing students to focus greater attention on coursework. Finally, our program structure brought together students of varied cultures and backgrounds who were able to share life experiences, build critical social and cultural bonds that helped to avert isolation, and enrich one another’s learning experiences. The combined aspects of our program worked to empower students to become self-regulated learners who can take greater control of their own medical education.

A common challenge for medical educators is how to best support previously enrolled students who need to repeat courses. Recognizing that our program may benefit such students, one year we were able to include them. These students had been offered but declined to participate in our program prior to entry to their first year due to work or other commitments. We found that these students were helped in much the same way as the students just entering medical school by facilitating the development of the skills and interaction with faculty while also expanding their peer support networks.

We further recognize that much of what the program participants gain would be beneficial to the larger student body. One challenge to implementing portions of the program to the first and second year curriculum is the required additional effort on the part of the school’s faculty. Further, many of the program activities are quite resource and space intensive which limits the ability to add more PBL and labs to the regular curriculum.

A concern that has been raised about programs such as ours that are designed for a limited number of disadvantaged students is that they may negatively impact the self-esteem of students if they perceive a message that they need special assistance to succeed in medical school [35]. This did not appear to be a problem among our students, however, we acknowledge that a limitation to this study is that it occurred at a single institution so some of our findings may not be generalizable. Many of our students were aware that their MCAT scores were lower than their peers, that their science background did not match that of their peers, or, most markedly among the Native American students, fully realized that moving to Minnesota was a big personal and cultural transition. In general, the participants have performed well; we are continuing to track outcomes and will report those data as numbers increase. We understand that it is not possible to deter all challenges a student will encounter in medical school, instead, our goal has been to empower students by engaging them to build their own skills, familiarizing with and showing them how to access resources, and build relationships so that they will possess the tools to overcome challenges. Future studies will be needed to determine the long-term effects on graduation of the program.

## Conclusions

Medical school is challenging for all students. For students from rural and Native American backgrounds these challenges can be magnified and, unfortunately, can result in delayed graduation and increased attrition. Institutional front-end efforts such as our pre-matriculation program can act as a starting point for students to develop skills and connections that feed educational persistence and allow them to flourish in their medical education.

## Appendix: Interview script for focus groups

**Questions for focus group**

1. Could you tell me about why you decided to participate in the pre-matriculation program? Did you have any other summer opportunities you had to give up to participate in the program?
2. What do you see as the benefits for students enrolled in this program?
3. What specific skills do you believe the program helped you to develop?
4. Before entering medical school, were you concerned about the medical school courses? Did participating in the pre-matriculation course affect any of these concerns?
5. Before entering medical school, were you concerned about being away from home/family? Did participating in the pre-matriculation course affect this?
6. How did your participation in the program affect your experience in the Foundations of Medicine course?
7. Are there additional ways the program could help facilitate your transition to the University of Minnesota Medical School?
8. Would you have preferred to participate in an online course rather than an in-person course?
9. In closing, our goal for this project is to learn about your experience with the pre-matriculation program. Given this, do you have any additional comments or information you would like to share as we plan for next year’s program?
